# Distinctive epigenomic alterations in NF1-deficient cutaneous and plexiform neurofibromas drive differential MKK/p38 signaling

**DOI:** 10.1186/s13072-020-00380-6

**Published:** 2021-01-13

**Authors:** Jamie L. Grit, Benjamin K. Johnson, Patrick S. Dischinger, Curt J. Essenburg, Marie Adams, Stacy Campbell, Kai Pollard, Christine A. Pratilas, Tim J. Triche, Carrie R. Graveel, Matthew R. Steensma

**Affiliations:** 1 Center for Cancer and Cell Biology, Van Andel Research Institute, 333 Bostwick Ave. NE, Grand Rapids, MI 49503 USA; 2 Center for Epigenetics, Van Andel Research Institute, Grand Rapids, MI USA; 3 Genomics Core, Van Andel Research Institute, Grand Rapids, MI USA; 4 Helen DeVos Children’s Hospital, Spectrum Health System, Grand Rapids, MI USA; 5 Department of Oncology, Sidney Kimmel Comprehensive Cancer Center At Johns Hopkins, Johns Hopkins University School of Medicine, Baltimore, MD USA; 6 Michigan State University College of Human Medicine, Grand Rapids, MI USA

**Keywords:** Epigenome, Neurofibromatosis, Neurofibroma, Inflammation, RAS

## Abstract

Benign peripheral nerve sheath tumors are the clinical hallmark of Neurofibromatosis Type 1. They account for substantial morbidity and mortality in NF1. Cutaneous (CNF) and plexiform neurofibromas (PNF) share nearly identical histology, but maintain different growth rates and risk of malignant conversion. The reasons for this disparate clinical behavior are not well explained by recent genome or transcriptome profiling studies. We hypothesized that CNFs and PNFs are epigenetically distinct tumor types that exhibit differential signaling due to genome-wide and site-specific methylation events. We interrogated the methylation profiles of 45 CNFs and 17 PNFs from NF1 subjects with the Illumina EPIC 850K methylation array. Based on these profiles, we confirm that CNFs and PNFs are epigenetically distinct tumors with broad differences in higher-order chromatin states and specific methylation events altering genes involved in key biological and cellular processes, such as inflammation, RAS/MAPK signaling, actin cytoskeleton rearrangement, and oxytocin signaling. Based on our identification of two separate DMRs associated with alternative leading exons in *MAP2K3,* we demonstrate differential RAS/MKK3/p38 signaling between CNFs and PNFs. Epigenetic reinforcement of RAS/MKK/p38 was a defining characteristic of CNFs leading to pro-inflammatory signaling and chromatin conformational changes, whereas PNFs signaled predominantly through RAS/MEK. Tumor size also correlated with specific CpG methylation events. Taken together, these findings confirm that *NF1* deficiency influences the epigenetic regulation of RAS signaling fates, accounting for observed differences in CNF and PNF clinical behavior. The extension of these findings is that CNFs may respond differently than PNFs to RAS-targeted therapeutics raising the possibility of targeting p38-mediated inflammation for CNF treatment.

## Introduction

Approximately 1 in 3000 live births are affected by the tumor predisposition condition, Neurofibromatosis Type 1 (NF1) making it the most common single gene-inherited condition in humans. One of the clinical hallmarks and diagnostic sine qua non of NF1 is the formation of benign peripheral nerve tumors called neurofibromas. Neurofibromas are a major cause of disfigurement, pain, and morbidity in NF1. These issues are predominantly attributable to two types of neurofibromas: dermal or cutaneous neurofibromas (CNFs) that develop in the skin, and plexiform neurofibromas (PNFs) that arise from nerves situated in deeper anatomic compartments. Even though CNFs and PNFs share similar histology, they are pathophysiologically distinct. NF1-related PNFs are associated with the development of high-grade malignant peripheral nerve sheath tumors (MPNSTs), which are the leading cause of death of NF1 patients [1]. In contrast, CNFs exhibit slower growth rates and do not have malignant potential [2–4]. Although CNFs do not become malignant, these benign tumors are often painful and disfiguring. These critical distinctions between CNFs and PNFs are not explained by genomic and transcriptomic studies that failed to identify consistent alterations in these tumors [5].

*NF1* loss of heterozygosity is thought to be the initiating event in CNF and PNF formation, making *NF1* the most commonly mutated gene in these tumors. Other genetic alterations have also been described but are inconsistent between and across tumor types. Previous genomic studies observed that approximately one third of CNFs exhibit focal chromosomal imbalance; however, there were no consistent chromosomal microstructure alterations among the samples analyzed [6]. Expression profiling studies have yielded mixed results as to whether CNFs and PNFs can be distinguished based on gene expression alone [5, 7]. It is important to note that these studies were not powered specifically to address differences between CNFs and PNFs, but rather focused on distinguishing PNFs and MPNSTs. Even though CNFs and PNFs arise in distinct anatomic sites, mouse models have demonstrated that CNFs and PNFs share a common cell of origin in Schwann cell progenitors (i.e., skin-derived precursor (SKP) [8] or GAP43 + PLP + precursors) [9]. Currently, it is not well understood how tumor microenvironment impacts neurofibroma progression [10], however inflammation from *NF1* haploinsufficient mast cells is requisite for PNF development [11]. Prior work examining epigenetic modifications in CNFs and PNFs has also been confounded by the lack of tissue- and patient-matched controls, which are not always available in the context of *NF1 *deficiency. Thus, there are insufficient data to adequately explain why CNFs and PNFs exhibit such distinct clinical behavior.

In this study, we performed genome-wide DNA methylation profiling using the Illumina Infinium MethylationEPIC BeadChip platform on a large discovery cohort of CNFs and PNFs. We demonstrate that CNFs and PNFs maintain epigenetically distinct profiles that are distinguishable based on both site-specific and chromosome-wide methylation differences. This work stands in contrast to prior studies that failed to identify differential methylation at the *NF1* locus in peripheral nerve sheath tumors, and were underpowered to identify signaling impacts based on genome-wide methylation differences [12][12]. Our work confirms that broad and distinct patterns of methylation result in differential signaling between CNFs and PNFs, as well as in the regulation of tumor size. Specifically, two differentially methylated regions (DMRs) in the *MAP2K3* and an upstream regulatory site for *MAPK14* were significantly altered between CNFs and PNFs leading to increased MKK3 and p38 expression. This epigenetic reinforcement of MKK3 and p38 expression also led to activation of both p38 and ERK based on an arbitrary subset of CNF patient samples within the discovery cohort. The MKK3/ p38 signaling axis is linked to both inflammation and pain signaling, as well as chromatin conformational changes through SWI/SNF complex regulation. ERK activation is not a usual consequence of MKK3/p38 activation due to conserved inhibitory feedback pathways. Taken together, our data confirm that epigenetic regulation of key RAS signaling genes results in disordered growth, inflammation and signaling in the context of NF1 deficiency. These findings also confirm the importance of epigenetic regulation in CNF tumor initiation and progression, as well as a potentially druggable signaling axis in MKK3/p38/ERK.

## Results

### CNFs and PNFs have distinct global methylation profiles

**Fig. 1:**
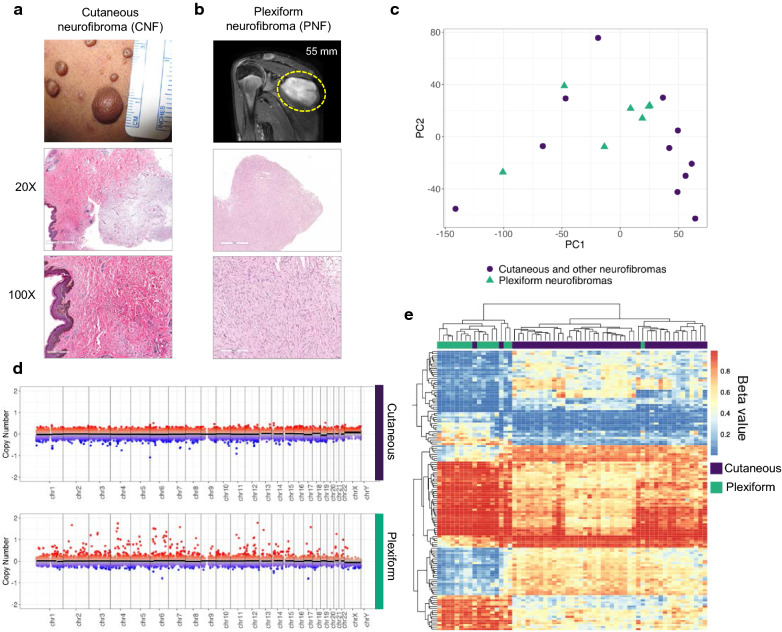
CNFs and PNFs are epigenetically distinct tumor types. **a** Clinical presentation of a CNF with H/E stain (20X/100X magnification shown); versus **b** PNF resected from deep shoulder location (T2-weighted MRI image; H/E 20X/100X shown). **c** Principal component analysis of RNAseq expression data comparing CNFs (purple) and PNFs (green) neurofibromas. **d** Copy number analysis of CNFs versus PNFs. **e** Unsupervised hierarchical cluster analysis of methylation probesets (i.e., beta-values) demonstrating unique CNF and PNF methylation profiles

CNFs are benign, skin-based neoplasms. These tumors typically arise in puberty and increase in number throughout adulthood. NF1 patients can develop between several to thousands of CNFs that cover significant portions of the body. Even though the size of CNFs can vary in NF1 patients (Fig. 1a), CNFs are associated with limited growth potential (typically < 3 cm). The primary treatment option for CNFs is surgical removal, however they are prone to recurrence and surgical treatment is impossible in patients with severe tumor burden. CNFs are composed of neoplastic Schwann cells, mast cells, and fibroblasts (Fig. 1a, middle and lower panels). In addition, CNFs often include a collagenous and myxoid extracellular matrix. CNFs and PNFs share nearly identical histology and require clinical context to accurately diagnosis [14]. In contrast, PNF growth is dysregulated, can develop anywhere in the body, and typically extends along peripheral nerves. Because of these features, PNFs can envelop organs and cause pain, disfigurement, and morbidity. For example, a PNF included in this study was removed due to its rapid growth in an axial location (Fig. 1b). Importantly, CNF-associated malignancies are rare, whereas PNFs are able to dedifferentiate into aggressive sarcomas in 8–13% of patients [15].

In order to confirm whether CNFs and PNFs are genomically and transcriptomically distinct lesions using advanced sequencing techniques, we compared publicly available RNA-seq data from an NF1 test cohort where cutaneous/non-plexiform neurofibromas were compared to PNFs [16]. Principal Component Analysis (PCA) of the RNA-seq data confirmed that histologically   similar cutaneous CNFs and PNFs cannot be discriminated based on global gene expression profiles, largely due to transcript heterogeneity (Fig. 1c). Prior expression profiling studies using cDNA microarrays did not identify distinct CNF and PNF transcriptome signatures at the macro-level, even though individual differences in key gene expression were identified [5]. We also evaluated whether genomic differences were present among our CNF and PNF cohorts and did not observe any large-scale differences in copy number variation (Fig. 1d). However, we note that there do appear to be focal copy number alterations identified in both CNF and PNF patient samples, consistent with observations from prior work (Fig. 1d, Supplemental Fig. 7) [17]. These results verify that few distinctive genomic or transcriptome alterations exist outside of putative *NF1* deficiency (Fig. 1c, d).

DNA methylation changes play key roles in development, disease and aging, with the added benefit of being a highly stable epigenetic mark that can be readily assayed, making this a clinically actionable and functional readout [18–22]. We determined neurofibroma methylation profiles using the Infinium MethylationEPIC array in a discovery cohort of 61 CNF and PNF samples from a total of 39 patients (Additional file 6: Table S1). The MethylationEPIC array is a targeted approach to interrogate approximately 850,000 methylation sites throughout the genome, including coding and regulatory space (e.g., FANTOM5 annotated enhancers) in addition to CpG islands, shores, and shelves. We applied a hierarchical generalized linear mixed effects model to identify differentially methylated loci between CNFs and PNFs, controlling for age and sex differences with a nested random effect to control for partially repeated measures. In total, we identified 31,201 significant differentially methylated probes (DMP; *q* < 0.05). By examining differentially methylated loci with an absolute odds ratio greater than 4 and clustering using a semi-supervised hierarchical approach, we establish a distinct, base-pair resolution epigenetic signature for CNF and PNFs (Fig. 1e). This probe-based analysis confirms that individual CpG-based methylation events are highly consistent within CNF and PNF tumor types, yet between CNFs and PNFs there are clearly definable, distinct global methylation profiles with minimal overlap. Comparison of beta-values within clustered methylation sites revealed striking differences across large portions of the genome suggesting that chromatin architecture is also distinct between tumor types.

### CNFs and PNFs display distinctive 3D chromatin architecture

**Fig. 2:**
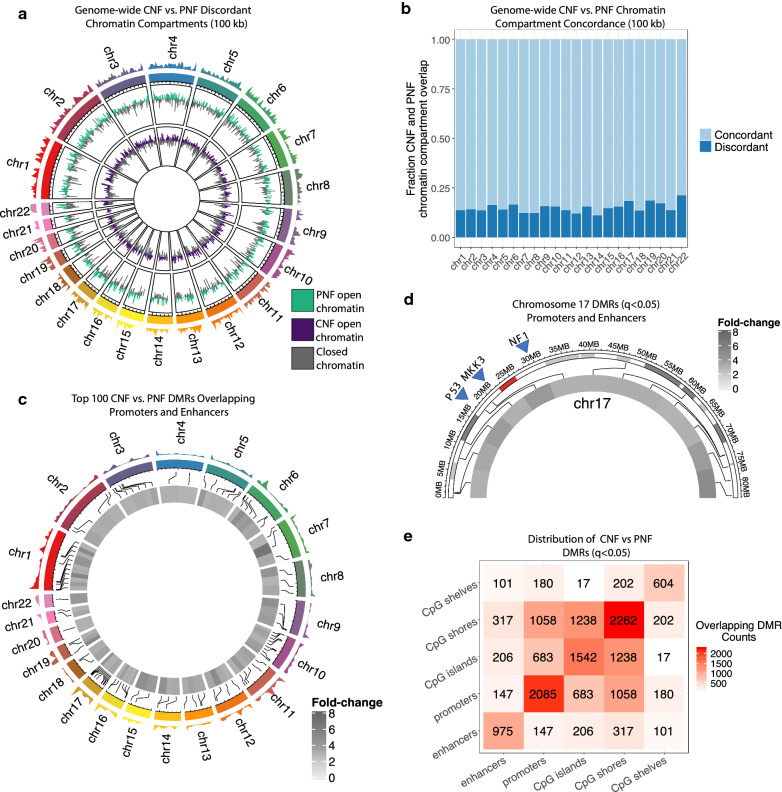
Chromatin architecture analysis reveals distinct structural and site-specific methylation events. **a** Genome-wide chromatin compartment calling (X-chromosome excluded) comparing CNFs to PNFs. b DMR analysis of overlapping promoter and enhancer space between CNFs and PNFs. Top 100 significant DMR sites shown. **c** Chromatin compartment concordance is depicted as the fraction of CNF and PNF compartment overlap at 100 kb resolution. **d** Chromosome 17 was the most differentially methylated chromosome. Chromosome-specific analysis of promoter and enhancer space shown. Red bar indicates position of the *NF1* gene. **e** Chromatin structural analysis demonstrating the distribution of DMR calls in topographic CpG categories (i.e., shelves, shores, islands, promoters and enhancers)

Next, we sought to determine if nuclear organization and higher-ordered chromatin states differed between CNFs and PNFs. Chromosomal DNA is organized into A/B compartments that largely correspond to being either transcriptionally active DNA compartments (A compartment) or silenced DNA compartments (B compartment) [23]. As such, DNA compartmentalization is a critical determinant of gene expression leading to cell fate determination and coordinated tissue development [24, 25]. Typically, chromatin compartments are identified using HiC or other assays to directly measure long-range chromatin contacts [26]. However, it has been demonstrated that Infinium Human Methylation 450K or EPIC array can reconstruct higher-order chromatin structure similar to HiC [27]. Thus, we inferred A/B group-level compartments in CNF and PNFs at 100 kb resolution. We show that the 3D genomic organization between CNFs and PNFs is 80–85% concordant whereas 15–20% of the compartments are inverted or discordant between CNF and PNFs. This discordance of PNF and CNFs compartments is observed genome-wide (X chromosomes were not assessed) (Fig. 2a, b). These data provide additional support that CNFs and PNFs possess distinct epigenomes. Given that individual CpG methylation changes and associated effects are difficult to interpret in isolation unless they are placed in the context of neighboring CpG loci and summarized into region-level changes, we used the differentially methylated probes identified above to call 6,004 significantly differentially methylated regions (DMRs; *q* < 0.05) using DMRcate. Using an ad hoc threshold of two-fold changes, a total of 1655 differentially methylated regions were significantly different (*q* < 0.05) between CNF and PNFs (Supplemental Fig. 6a). A focused evaluation of the top 250 DMRs in terms of fold-change (Supplemental Fig. 6b) revealed that 100 of these sites were found to occur in the genomic contexts of promoter and enhancer space, providing evidence that these DMRs may play a regulatory role in modulating gene expression (Fig. 2b). Notably, we observed that areas of promoter/enhancer overlap were evenly distributed in these top differentially methylated regions across all chromosomes except chromosome 17 which was disproportionately affected (Fig. 2c, d). Chromosome 17 contained 13 top DMRs, while other autosomal chromosomes contained on average, 3.95 ± 2.20 DMRs. Intriguingly, these DMRs spanned the entire length of Chromosome 17, including putative regulatory space of key genes such as *NF1*, *TP53,* and *MAP2K3* (Fig. 2d). Taking into account all significant DMRs (q < 0.05), genomic context distributions revealed a broad distribution that extended beyond the promoter and enhancer space, including CpG islands, shores, and shelves (Fig. 2e), in line with the features targeted by the Illumina EPIC methylation arrays. Taken together, these results provide evidence that *NF1 *deficiency in transcriptionally and genomically similar lesions possess distinct epigenomes as evidenced through genome-wide differences in higher-order chromatin structure and differentially methylated regions. These results link *NF1 *deficiency with differential methylation events that likely alter signaling fates through epigenetic alterations in regulatory space and culminates in broad remodeling of chromatin architecture throughout the genome. Given the lack of transcriptional and genomic differences between CNF and PNFs, these data strongly suggest that epigenomic differences contribute to the morphological and pathological variation observed in NF1 patients.

### CNF size variation is correlated with differential methylation

**Fig. 3:**
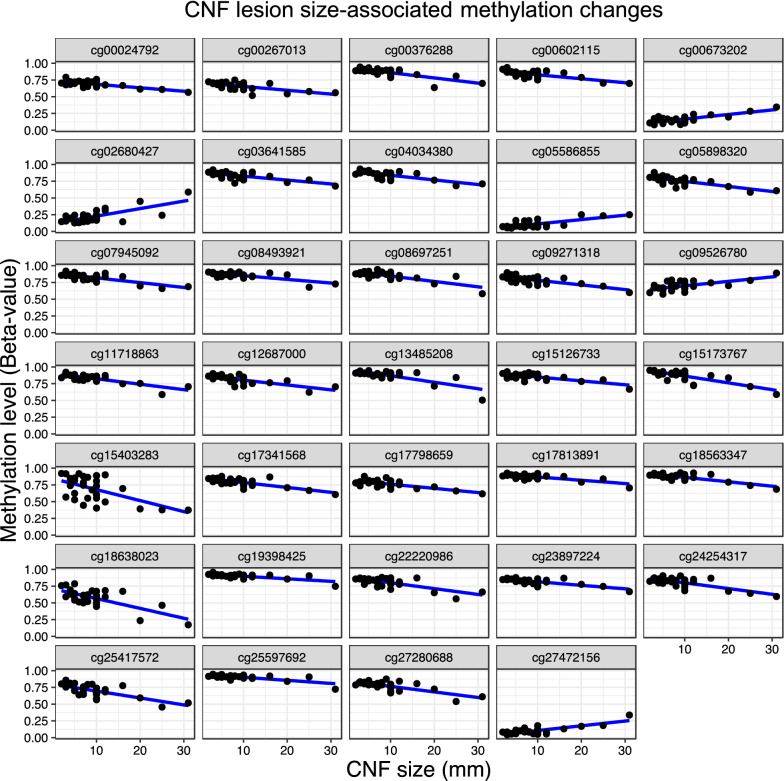
Differential methylation correlates with CNF tumor size. Graphical comparison of methylation levels (i.e., beta-values) versus CNF size (mm) at DMRs determined to be significantly correlated with measured size of CNF tumors

The degree of CNF involvement is highly variable among NF1 patients with poor genotype–phenotype correlation. CNF density is even noted to be discordant among monozygotic twins [28]. To determine whether CNF size correlates with site-specific methylation events, we collected CNFs in three size categories. Adjusting for age and sex differences, we determined significant associations (*q* < 0.05) between CNF size in millimeters and probe-level methylation status. A total of 188 loci were found to be statistically significant. Both positive and negative correlations were discovered among the 188 associated loci, with 34 achieving an effect size (> 4 mm) that reached statistical significance (Fig. 3). Signaling pathway correlation of the 34 significant DMRs was not possible due to a lack of statistical confidence, however these results indicate that CNF size is significantly influenced by methylation of specific loci.

### Differentially methylated loci are enriched in inflammatory pain and RAS pathways

**Fig. 4:**
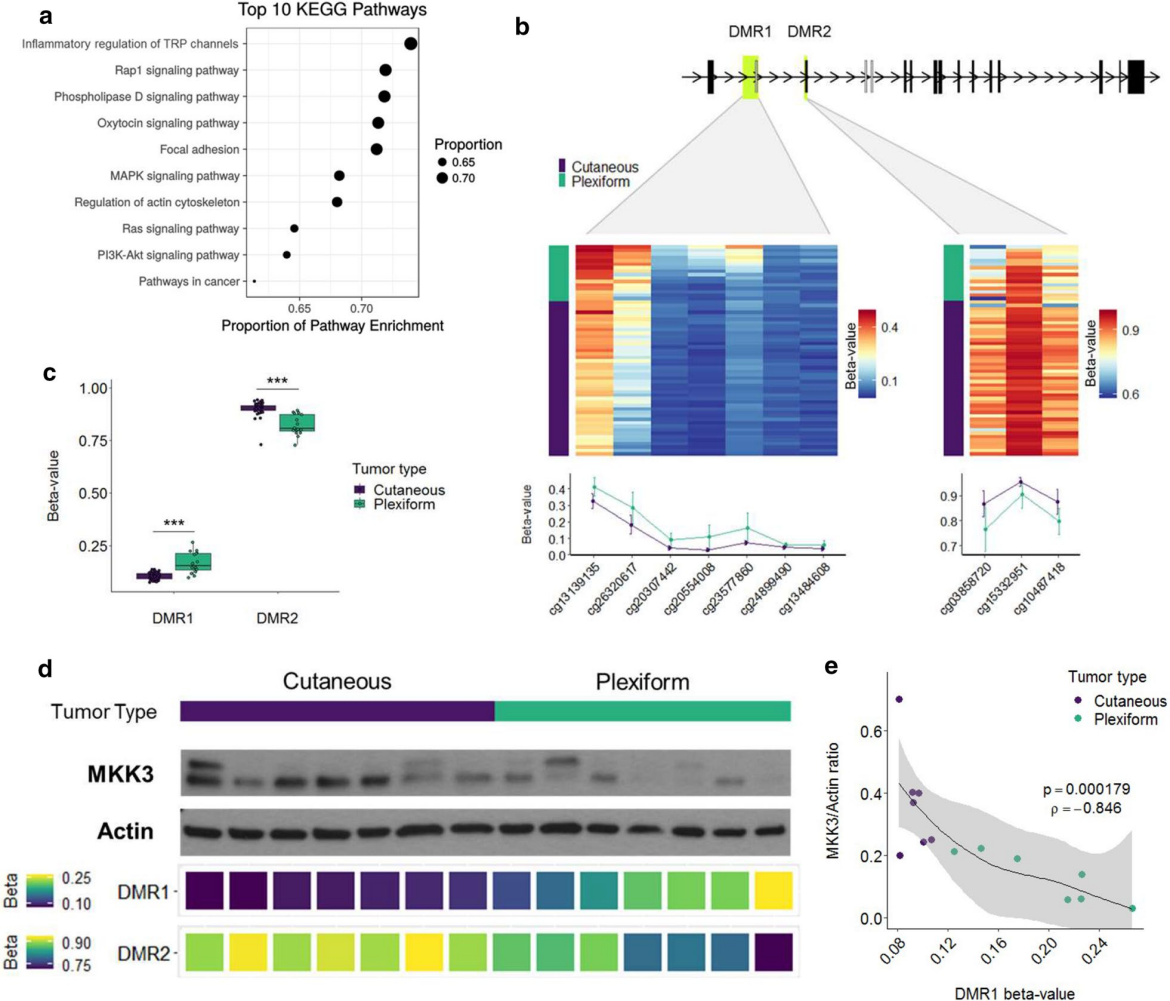
Functional annotation of significant DMRs reveals differential methylation at alternative *MAP2K3* exons leading to coordinated MKK3 expression. **a** KEGG pathway analysis of CNF and PNF methylation profiles confirms that multiple pathways are regulated epigenetically including growth factor, inflammatory, pain signaling and oxytocin pathways. **b**, **c** Differential methylation was observed in upstream regulatory sites in *MAP2K3* that control alternative leading exon expression. **d** Western blot analysis of MKK3 protein (actin loading control). Beta-values for tumor-type DMRs (DMR1/DMR2) shown below. **e** DMR1 beta-values correlate strongly with MKK3/Actin ratio expression values (*p* < 0.01; = − 0.846)

To understand how the distinct methylation patterns may promote CNF and PNF development, we performed a gene ontology analysis to determine whether DMR patterns would predict signaling in critical pathways. Kyoto Encyclopedia of Genes and Genomes (KEGG) pathway enrichment analysis is a high-level functional analysis of epigenomic data that allows for annotation of key cell signaling and biological processes. KEGG analysis of global DMR data identified several key cellular processes that differed significantly between tumor types as a result of predicted gene expression impacts from methylation events. As expected, the RAS, MAPK, and PI3K–AKT signaling pathways were associated with both CNF and PNF development; however, differential methylation was noted between the tumor types in genes affecting key cellular processes. Interestingly, CNFs and PNFs demonstrated differential regulation of inflammatory mediators of transient receptor potential (TRP) channels, phospholipase D (PLD), cytoskeletal rearrangement, and oxytocin signaling (Fig. 4a). TRP channels, PLD, and oxytocin signaling have all been demonstrated to regulate inflammation and pain, which is a common feature of both PNFs and CNFs. Pain and inflammation are known to be dysregulated in Neurofibromatosis, however it is unclear how NF1 deficiency exacerbates these symptoms. To functionalize these results and validate a role for individual DNA methylation events in tumor-type associated inflammation and pain signaling, we examined significantly correlated DMRs associated with genes in the Inflammatory Mediator Regulation of TRP Channels KEGG pathway. Two highly significant DMRs (DMR1 *p* = 2.72E-21; DMR2 *p* = 1.84E-08) were discovered within the primary and alternative promotors and leading exons of the *MAP2K3* gene that encodes the MAP kinase kinase, MKK3 (Fig. 4b). Across DMR1, PNFs displayed higher DNA methylation, while CNFs were more highly methylated in DMR2 (Fig. 4c). As MKK3 plays a role in pain signaling, inflammation and cancer [29, 30], we hypothesized that reciprocal methylation of DMR1 and DMR2 would instruct differential MKK3 protein expression leading to altered RAS signaling fates. Western blot of a representative subset of CNF and PNF tumors demonstrated that MKK3 protein expression was highly correlated to *MAP2K3* DMR methylation status (DMR1:MKK3 *ρ* = − 0.85, *p* = 0.00018) (Fig. 4d, e) despite expected intra- and inter-tumoral heterogeneity. This strong inverse correlation points to exquisite control of MKK3 protein expression through methylation events occurring at sites of alternative leading *MAP2K3* exons.

**Fig. 5:**
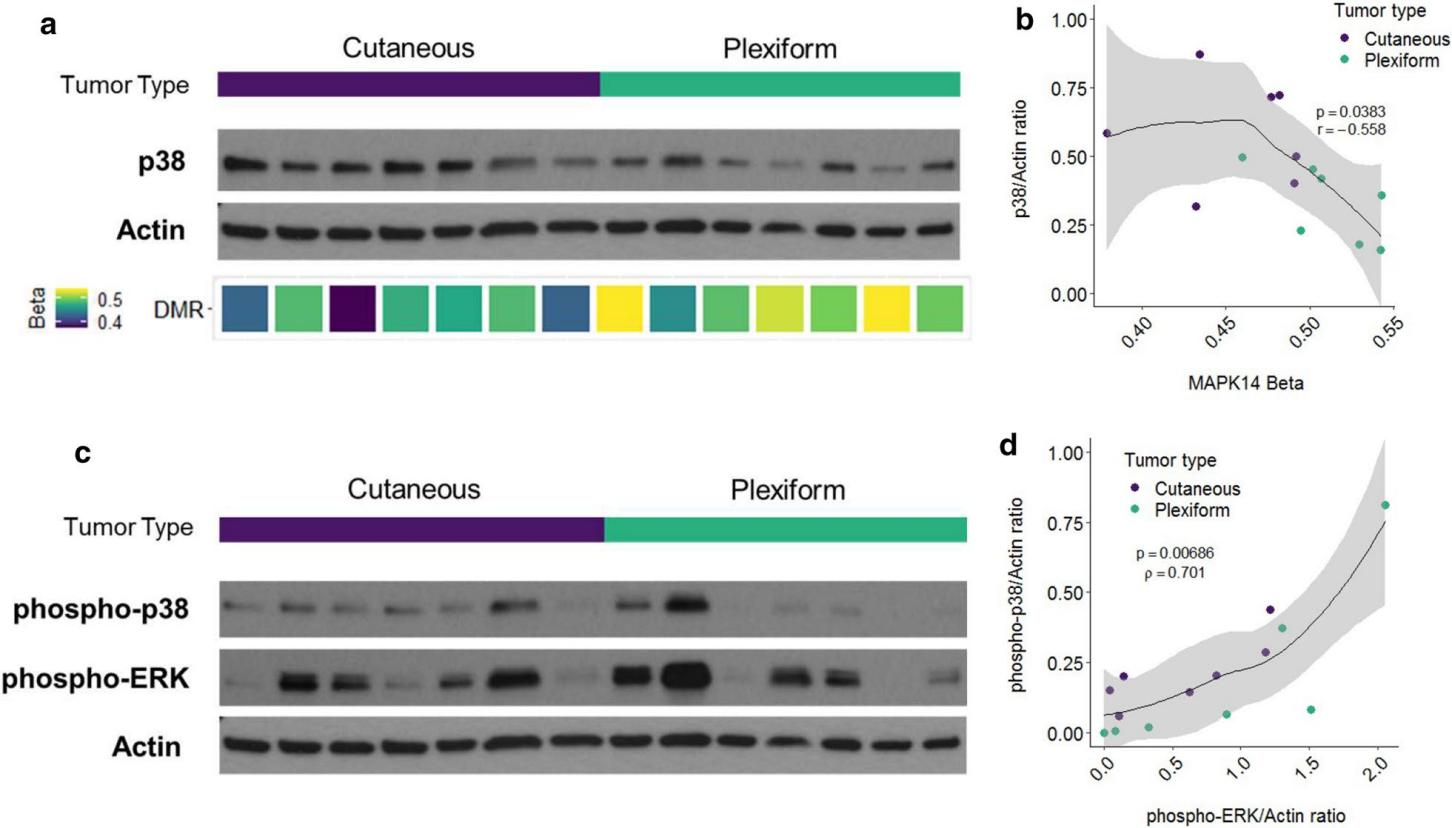
MKK3/P38 signaling axis is epigenetically reinforced through methylation events in critical regulatory regions. **a** p38 expression (WB) shown between CNFs and PNFs. P38 expression strongly correlates with **b** promoter methylation in *MAPK14* (beta-values shown; *p* = 0.038; = − 0.6). **c**, **d** Phospho-p38 expression and phospho-ERK expression are demonstrated by western blot (**c**) and were significantly correlated (**d**) (*p* = 0.006; = 0.70)

As p38 is the primary effector of MKK3 signaling in response to cellular stress and cytokine stimulation [31], as well as chromatin conformational changes such as those observed in Fig. 2, we hypothesized that DNA methylation also regulates p38 expression in CNFs and PNFs. Because methylation events can also impact protein kinase activation states [32], we also assessed whether *MAP2K3* methylation events affected downstream p38 protein expression and activation. We identified a significant DMR (*p* = 1.66E−16) approximately 3.5 kb upstream of the *MAPK14* gene, which encodes the p38α isoform. Although the DMR lies within the promotor region of *SLC26A8* gene, p38 protein levels were negatively correlated with methylation at this site (ρ = − 0.56, *p* = 0.038) (Fig. 5a,b). Methylation was higher in the PNF group than the CNF group leading to variable but overall decreased p38 protein expression (T180/T182) in PNFs (Fig. 5a). More specifically, CNFs expressed both MKK3 (Fig. 4d) and p38 (Fig. 5a) more abundantly than PNFs, as well as phospho-p38 indicating consistent p38 activation (Fig. 5c). These results are consistent with canonical MKK3/P38 pathway activation. Whereas MKK3 maintains a high degree of specificity for p38, it is unclear whether p38 regulates ERK through direct crosstalk [33]. P38 activation was previously shown to inhibit RAS through a redundant negative feedback loop originating in downstream MAPK signaling [34]. It is currently unclear how NF1 deficiency affects negative feedback mechanisms downstream of RAS. We demonstrate that p38 activation was strongly correlated with pERK expression regardless of tumor type (*ρ* = 0.70, *p* = 0.006) (Fig. 5d). These results point to discrete signaling differences between RAS/MEK/ERK and RAS/MKK3/ p38/ERK in the context of NF1 deficiency (Fig. 5c, d).

## Discussion

This study represents the most comprehensive epigenetic analysis of CNFs and PNFs to date. Greater than 99% of NF1 patients exhibit both CNFs and PNFs over the course of their lifetime accounting for a substantial negative impact on quality of life [4]. Pain is a constant feature of both neurofibroma subtypes, yet how pain signaling occurs in peripheral nerve tumors is poorly understood. Despite the recent demonstration of MEK inhibitor effectiveness in PNF treatment [35], it is unclear whether CNFs respond with equal efficacy. More therapies are needed to treat neurofibroma tumor progression and symptoms, such as pain and itching, as all of these clinical features contribute significantly to morbidity in NF1. Our findings address an unmet clinical need for neurofibroma treatment and offer significant mechanistic insight into how benign nerve tumors initiate, progress, and generate symptoms through epigenetic means. It is now conceivable that NF1-deficient neurofibromas can be treated by targeting epigenetic mechanisms that reinforce RAS signaling and its association with inflammation.

In the absence of distinctive transcriptomic or genomic alterations in CNFs and PNFs apart from putative NF1 deficiency, our work confirms that methylation events are key molecular determinants of nerve tumor initiation, growth, and pain generation. How epigenetic regulation of kinase signaling affects cancer predisposition in these tumors remains unclear; however, recent data confirms that accumulating epigenetic alterations in a single field or region are associated with elevated cancer risk [36–40]. Thus, our data lay the groundwork for future studies examining how epigenetic alterations affect PNF conversion into MPNSTs, as well as protective mechanisms that spare CNFs from cancerous progression or unchecked tumor growth. The identification of robust differences in the methylation profiles of CNFs versus PNFs also confirms their distinct biology, as well as laying the groundwork for future development of clinical biomarkers.

Chromatin conformational states differed significantly between CNFs and PNFs and were strongly linked to both site-specific and geographic-specific methylation events. These findings suggest that chromatin accessibility broadly affects gene expression in CNFs and PNFs. More work is needed to determine how epigenetic alterations affect regulatory genes that are known to contribute to tumor size and, ultimately, cancer predisposition. Based on our probe-based analysis of tumor tissue, we identified 34 CpG methylation sites that were statistically correlated with CNF size. Unfortunately, the genes corresponding to the individual methylation probe sites could not be identified with statistical confidence, nor could we link these methylation events with specific biological processes or signaling pathways. Regardless, these data confirm that CpG methylation influences CNF tumor size, possibly through a novel mechanism. More work is needed in this area.

Our data confirms that CNFs and PNFs strongly exhibit differential methylation at two established DMRs (i.e., DMR1 and DMR2) that are situated immediately upstream of the *MAP2K3* transcriptional start site. This pattern of differential methylation resulted in upregulated protein expression of MKK3 and p38 in CNFs, whereas in PNFs the reciprocal effect was observed with downregulated expression (Fig. 5). This effect was consistent within and across tumor types despite expected signaling heterogeneity from analyzing whole tumor tissue from multiple subjects. The cell types that contributed to the observed differences in methylation profiles could not be determined. Unfortunately, deconvolution analysis is dependent on cell type-specific profiles which are lacking for NF1-deficient neurofibromas.

*MAP2K3 *was previously identified as a candidate imprinted gene in the context of NF1 deficiency [41], but the roles of its upstream DMRs are not well characterized. DMR1 is generally thought to regulate gene expression of an alternative coding region with sequence homology to exon 1, whereas DMR 2 regulates exon 1 expression directly. The importance of alternative exon expression in cancer is increasingly being recognized as it has been used to identify breast cancer subtypes using RNAseq data from the The Cancer Genome Atlas (TCGA) Breast Invasive Carcinoma (BRCA) cohort [41]. DNA methylation status was also shown to affect expression of alternative exons in the sphingosine 1-phosphate (SPHK1) gene in gastroesophageal cancer [42, 43]. Apart from these studies, the impact of alternative exon expression on tumorigenesis has not been well described, nor has the role of methylation in defining which *MAP2K3* exon is preferentially expressed.

**Fig. 6:**
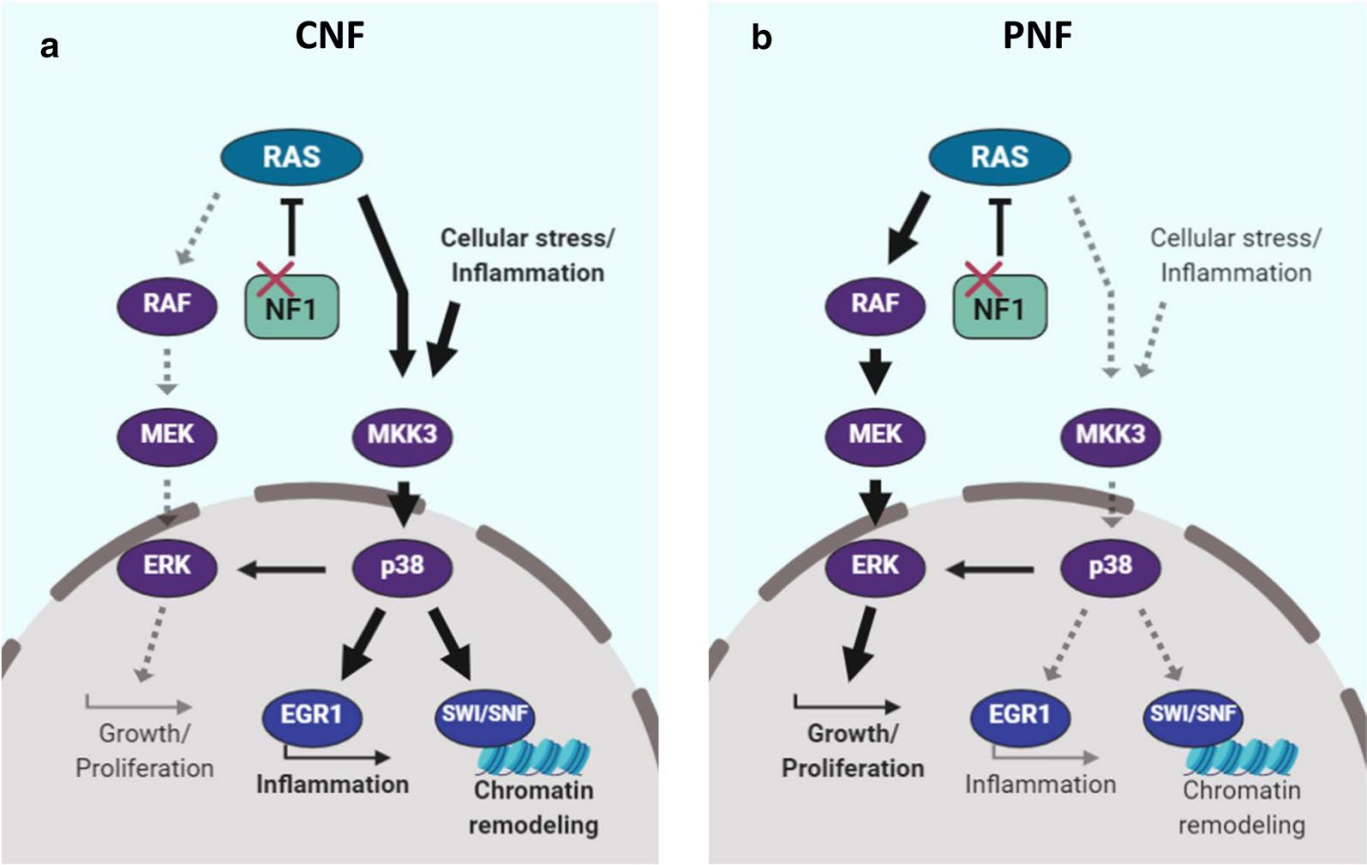
Proposed differential signaling schema comparing PNFs and CNFs. Although both RAS/MEK/ERK and RAS/MKK3/P38 signaling occurs in both tumor types, RAS activation results in differential signaling strength and fidelity through RAS/MEK in PNFs (left panel) and, alternatively, RAS/MKK3/P38 in CNFs (right panel). CNFs divert RAS activation through MKK3 thereby enhancing the cellular response to stress and inflammation that is mediated by MKK3/p38, whereas the downstream impact in RAS/MEK dependent PNFs is unchecked growth and proliferation (left). CNFs still maintain growth and proliferation signaling through RAS/ERK or p38/ERK, but strongly exhibit a pro-inflammatory phenotype and are characterized by broad chromatin remodeling (right)

Our work extends these important findings by identifying alternative exon utilization as a potential epigenetic regulatory mechanism for the MKK3/ p38 signaling axis. MKK3/p38 is a critical pathway that couples RAS-mediated growth and proliferation with inflammation (e.g., EGR1) and chromatin remodeling (e.g., SWI-SNF). More broadly, these data strongly point towards epigenetic control of RAS signaling fates downstream of NF1. We propose a schema where p38 activation in response to cellular stress and cytokine signaling inputs is reinforced in CNFs, whereas PNFs appear to signal predominantly through RAS/MEK/ERK leading to growth and proliferation (Fig. 6, schematic). Future studies are needed to better define the implications of p38 activation in neurofibromas and their various cellular constituents. It is important to note, however, that crosstalk between the MKK3/p38 and RAS/MAPK signaling pathways has not been extensively studied. Prior work suggests a potential inhibitory role for RAS/ERK in mitigating p38-mediated inflammation [44]. Interestingly, p38 is not typically activated in response to mitogenic stimuli, but we observed a high degree of correlation between phospho-p38 and phospho-ERK expression. These results suggest that differential methylation may enhance crosstalk between MKK3/p38 and RAS/ERK leading to mixed signaling effects in the context of NF1 deficiency.

Proof of this concept comes from our observation that upregulated MKK3 protein expression, in turn, correlated with both p38 protein expression (p38) and activation (phospho-p38) indicating strong epigenetic reinforcement of the MKK3/p38 axis in CNFs (Fig. 4). Two expected results of p38 activation are activation of the MKK3/p38/EGR1 inflammatory cascade [45] and changes in chromatin conformation mediated through the SWI-SNF complex family [46]. Relevant to the role of EGR1 in the pro-inflammatory response, it is intriguing that in our unbiased gene set enrichment analysis we identified inflammatory mediator regulation of TRP channels and phospholipase D signaling as the most significant altered signaling pathways related to DMRs (Fig. 4a), granted EGR1, itself, was not found to be differentially methylated (data not shown). Pain is a constant feature of CNFs and PNFs leading to significant morbidity. Pain signaling in nerve tumors is not well understood and is difficult to manage. These data identify a potentially novel mechanism for epigenetic regulation of pain signaling in nerve tumors and a targetable signaling axis in MKK3/p38.

p38 is involved in the direct recruitment of SWI/SNF complexes to gene promoters resulting in chromatin modification and enhanced expression [47]. Although the methylation states of SWI/SNF complex family member DMRs were not discordant between CNFs and PNFs, it is plausible that reinforced MKK3/p38 signaling would exert its effect through SWI/SNF leading to the observed conformational changes. Further studies are needed to determine how SWI/SNF affects expression of genes involved in growth, proliferation, and inflammation. Moreover, the effects of targeting p38 may be amplified by expected loss of recruitment of SWI/SNF complexes to target genes.

## Conclusion

The epigenetic distinctions between CNFs and PNFs extend from the level of chromatin conformational change down to altered expression of proteins that regulate or modulate RAS signaling. These findings are intriguing given that the analyzed tumors arose in the context of RAS deregulation as a result of NF1 deficiency. Based on KEGG pathway analysis, it is likely that methylation events are involved in regulation of pain signaling down to the level of inflammatory mediator production. More work is needed in many aspects of neurofibroma epigenetics, including studies targeting p38 and its downstream effectors. As such, we present a new signaling paradigm where differential methylation between tumor types results in reinforcement of inflammatory signaling in CNFs, and classical RAS/MEK/ERK activation towards growth in PNFs.

## Methods

### Trial participants and sample collection

45 cutaneous neurofibromas, 17 plexiform neurofibromas, and 9 normal skin and nerve samples were collected from individuals with a confirmed diagnosis of Neurofibromatosis Type 1. These samples were collected prospectively under an approved Spectrum Health/Van Andel Research Institute IRB protocol (SH/VAI IRB#2014–295) (NCT02777775). Additional specimens were analyzed according to ethical standards and under a Johns Hopkins Hospital (JHH) institutional review board (IRB)-approved protocol (JH IRB # J1649, PI Pratilas). The JH NF1 biospecimen repository is supported by a grant from the Neurofibromatosis Therapeutic Acceleration Program (NTAP, n-tap.org), to C.A.P. Analysis by Sage Bionetworks is supported through the Neurofibromatosis Therapeutic Acceleration Program (NTAP, n-tap.org). Informed consent was obtained from all participants. Tumor samples were isolated by microdissection to remove adjacent normal tissue then snap frozen in liquid nitrogen and stored at − 80 °C. Quality parameters included assessment of percent content (> 95%) and viability (> 90% nuclear viability) by H/E staining. Biospecimen handling was performed according to BRISQ guidelines.

### 5mC interrogation by Infinium MethylationEPIC array

To extract DNA, frozen tissue was manually dissected into small pieces and homogenized by bead beating (Lysing Matrix D; MP Biomedicals) in UltraPure phenol:chloroform:isoamyl alcohol (ThermoFisher) according to the manufacture’s protocol. DNA was quantified by Qubit fluorometry (Life Technologies) and 500 ng of DNA from each sample was bisulfite converted using the Zymo EZ DNA Methylation Kit (Zymo Research, Irvine, CA USA) following the manufacturer’s protocol using the specified modifications for the Illumina Infinium Methylation Assay. After conversion, all bisulfite reactions were cleaned using the Zymo-Spin binding columns and eluted in 12 uL of Tris buffer. Following elution, BS converted DNA was processed through the EPIC array protocol. The EPIC array contains > 850 K probes querying methylation sites including CpG islands and non-island regions, RefSeq genes, ENCODE open chromatin, ENCODE transcription factor binding sites, and FANTOM5 enhancers. To perform the assay, 7uL of converted DNA was denatured with 1ul 0.4 N sodium hydroxide. DNA was then amplified, hybridized to the EPIC bead chip, and an extension reaction was performed using fluorophore-labeled nucleotides per the manufacturer’s protocol. Array beadchips were scanned on the Illumina iScan platform and probe specific calls were made using Illumina Genome Studio software.

### EPIC methylation array data pre-processing

Data were analyzed using a modified workflow that is similar to the ChAMP methylation array analysis procedure in R (v3.5.1). Briefly, samples were filtered for probes with poor or skewed intensities (detection *p*-value < 0.01) and entire samples were removed from the dataset if they contained > 10% failed probes. One sample exceeded the aforementioned filtering criteria and was removed from the dataset resulting in 70 total samples. Next, probes that have previously been identified to skew downstream differential methylation analyses (SNP probes, cross-reactive probes with other genomic regions, etc.) were removed in addition to probes that target sex chromosomes [48]. Next, a single-sample normalization method (ssNOOB) was applied to each sample to normalize and remove background signal from the data prior to downstream analyses (Supplemental Fig. 4) [49]. Sources of technical variation were found to significantly (*p* < 0.05) contribute to the variation explained in the first couple principle components in addition to tissue effects based on SVD analysis as implemented in ChAMP (v2.18.3) and corrected using the sva (v3.30.1) package in R for visualization purposes only. The results of this procedure for the first 11 PCs can be found in Supplemental Fig. 5. Technical variation was modeled as either fixed or random effects on the uncorrected data in the differential methylation analysis described below. Further, prior to differential methylation calling, additional SNP and CpH probes were removed using ChAMP (v2.18.3) using the default parameters for the function rmSNPandCH. In total, 717,148 probes were analyzed for differential methylation across tissue types.

### Differential methylation analysis

Differentially methylated loci between cutaneous and plexiform neurofibromas were identified using a hierarchical generalized linear mixed model (GLMM) approach with a logit link function as implemented in glmmTMB (v0.2.2) on the pre-processed beta-values. Partially repeated tissue sampling was modeled as a random effect with patient nested within methylation array slide unless specified otherwise. Group-level differences were determined using a likelihood ratio test (LRT) with a significance threshold of *q* < 0.05. False discovery rate adjustment was done using the Benjamini–Hochberg procedure. Models were filtered out for downstream analysis if they failed to converge. In total, 31,201 probes were found to be differentially methylated between cutaneous and plexiform neurofibromas. Differentially methylated regions were called using DMRcate (v1.18.0). Results from glmmTMB were wrangled into a suitable data structure as input for DMRcate by using the Wald statistic as the stat and a quasi-beta fold-change using the exponentiated model estimates for each probe. Default parameters were used for DMRcate with the bandwidth scaling factor (C parameter) set to 2. To identify differentially methylated loci associated with cutaneous neurofibroma size, a GLMM with a logit link was fitted, adjusting for age and sex differences and a random intercept term for partially repeated tissue sampling. Significant associations (*q* < 0.05) between CNF size in millimeters and probe-level methylation were determined as described above. A total of 188 loci were found to be significant. Positive or negative correlations were computed on significant probes using Kendall’s Tau. False discovery rate was controlled using the Benjamini–Hochberg procedure and significant correlations were determined at a *q* < 0.05 threshold. Significantly correlated probes were filtered on a delta Beta-value (maximum Beta-value minus the minimum Beta-value) of 0.2, resulting in 34 loci.

### Inference of chromatin conformation from EPIC methylation arrays

Chromatin compartments were computed at 100 kb resolution as previously described and implemented in compartmap (v1.65.71). Briefly, pre-processed M-values were subset to “open sea” CpG probes (at least 4 kb away from annotated CpG island) and masked probes that were found in at least 50% of samples were imputed using k-nearest neighbor via the impute (v1.59.0) R package. Next, loci were median summarized in 100 kb bins. Group-level compartments were inferred by computing Pearson correlations of summarized bins and the first principal component of the correlation matrix. A/B compartments correspond to positive (open chromatin) and negative (closed chromatin) eigenvalues, respectively. Genome-wide discordant compartments were identified by comparing the sign of the eigenvalue for overlapping genomic bins and filtering out those with small absolute eigenvalues (> 0.02). In total, we identified 2937 discordant chromatin compartments between plexiform and cutaneous neurofibromas. Results were plotted using circlize (v0.4.8).

### Pathway and GO term enrichment

Enrichment of pathways and gene ontology (GO) terms was performed using the gometh function within the missMethyl (v1.16.0) package in R. Briefly, gometh considers the relatively uneven density of loci covered on the Infinium methylation arrays and utilizes this information when computing enrichment, similar to the approach goseq uses for RNA-seq. Significant CpGs were identified as described above and the background probe set was derived following pre-processing. Significant GO terms and KEGG pathways were determined using a *q* < 0.05 threshold. Results were plotted using ggplot2 (v3.2.1).

### Tissue purity estimates

Tissue purity was estimated by PAMES (v0.2.3) and annotations built for computing informative sites using IlluminaHumanMethylationEPICanno.ilm10b2.hg19 (v0.6.0). Cutaneous neurofibromas were compared against normal skin samples and plexiform neurofibromas were compared against normal nerve. Results were plotted using ggplot2 (v3.2.1).

### Copy number estimation from EPIC methylation arrays

Copy number alterations were computed using SeSAMe (v1.3.2) with minor modifications for plotting functionality [50]. Briefly, raw data were processed using the “open SeSAMe” procedure, producing a signal set object. Next, samples were segmented (50 kb bins) and called for copy number differences, comparing CNFs to normal skin and PNFs to normal nerve using the cnSegmentation function with default parameters in SeSAMe. The genome annotation used was hg19 and relevant annotations are pulled in automatically from the sesameData package by the cnSegmentation function. Data were visualized using the log2 ratio of per-sample signal over the reference.

### RNA-sequencing data analysis

Paired-end, raw neurofibroma and plexiform neurofibroma RNA-seq data were downloaded from syn4939902. Sequencing lanes were merged, followed by alignment with STAR (v2.7.0f) to b37 (downloaded from the GATK resource bundle—https://software.broadinstitute.org/gatk/download/bundle), using the Gencode v19 annotations). Alignment was performed using default parameters with the following modifications: -twopassMode Basic, -outSAMtype BAM SortedByCoordinate, and -quantMode GeneCounts. Reverse-stranded gene counts were read into R (v3.6.1) using edgeR (v3.27.14), excluding sample 2-025 due to sample quality. Gene counts were restricted to known, protein coding genes and lincRNA. Samples were further filtered to genes that had greater than 1 count per million (CPM) in at least 3 samples. Libraries were normalized using the trimmed mean of M-values method. Dimensionality reduction was performed using the prcomp function (v3.6.1) on the filtered log2 CPM and plotted using ggplot2 (v3.2.1).

### Western blotting

For protein analysis, samples were divided into methylation high, methylation intermediate, and methylation low groups based on the beta-values across MAP2K3 DMR1 and samples from each group were randomly chosen as a representative subset of the discovery cohort. Protein lysates were prepared by manually homogenizing frozen tissue in RIPA buffer containing protease and phosphatase inhibitor cocktails (Roche). Proteins were separated on a 4–20% TGX SDS-PAGE gel (Bio-Rad) and transferred to a PVDF membrane (Invitrogen). Blots were blocked in 5% dry milk in TBST buffer (20 mmol/L Tris–HCl pH 7.4, 150 mmol/L NaCl, 0.1% Tween-20) and incubated at 4 °C overnight in primary antibody; MKK3 (Cell Signaling #5674), p38 (Cell Signaling #9219), phospho-p38 (Cell Signaling # 4511), phospho-ERK1/2 (Cell Signaling #9101), and β-Actin (Cell Signaling #3700). Densitometry was done in ImageJ.

### Data availability

Raw and processed EPIC array data are available from Synapse: syn4939910. Raw RNA-seq fastq data were downloaded from syn4939902.

### Statistical methods

Pairwise comparisons of beta-regressions calculated by betareg (v3.1–2) were done using emmeans (v1.4.2). For correlation analysis, normality was first assessed by a Shapiro–Wilk normality test. Data following a normal distribution were analyzed by Pearson’s product–moment correlation, while non-normally distributed data were analyzed by Spearman’s rank correlation rho. For all correlation plots, data were fit by stat_smooth using loess with span = 1 using ggplot2 (v3.2.1). All analyses were done using R (v3.6.1).

## Supplementary Information

**Additional file1**:** Figure S1**. Purity analysis CNF and PNF tissue. a) Cutaneous and plexiform neurofibroma methylation profiles are compared against normal controls. Purity estimates indicate significant enrichment of tumor tissue in queried samples. b) Size Comparison reveals equal purity among the three categories of tumors: incipient (<5mm), medium (5-10mm) and large (>10mm) .** Figure S2**. MAP2K3 is differentially methylated between PNFs and CNFs. a) Mean beta-values at individual positions within MAP2K3 DMR1 plotted by tumor type. Error bars reflect standard deviation. b) Individual beta-values within MAP2K3 DMR1 plotted by tumor type. Each line reflects a single tumor. c) Mean beta-values at individual positions within MAP2K3 DMR2 plotted by tumor type. Error bars reflect standard deviation. d) Individual beta-values within MAP2K3 DMR2 plotted by tumor type. Each line reflects a single tumor.** Figure S3**. Methylation of MAPK14 is increased in PNFs compared to CNFs. a) Mean beta-values at individual positions within MAP2K3 DMR1 plotted by tumor type. Error bars reflect standard deviation. b) Individual beta-values within MAP2K3 DMR1 plotted by tumor type. Each line reflects a single tumor.

**Additional file2**:** Figure S4**. Raw and normalized beta value densities from Illumina EPIC methylation arrays across patients and sample types. a) Raw beta value density plots following removal of failed samples and low-quality and cross-reactive probes (see Methods). b) Normalized beta value density plots following single-sample normalization (ssNOOB) reduces per-sample technical variation that can skew downstream analyses.

**Additional file3**:** Figure S5**. Singular value decomposition analysis of technical and biological sources of variation. Detection of sources of technical variation that need to be accounted for in the differential methylation models and adjusted for prior to visualization was accomplished using the champ.SVD function implemented in ChAMP. Technical factors such as slide and scan date were found to contribute significant sources of variation in the data in addition to biological factors, such as sex, that needed to be added as covariates in the differential methylation models to test for sample group differences (CNF and PNF).

**Additional file4**: ** Figure S6**. Volcano plots of top significantly differentially methylated regions. a) Significantly differentially methylated regions (DMRs; q<0.05) with a log2-quasi-fold change (see Methods) greater than 1 are highlighted with black dots. Gray dots correspond to DMRs falling below the log2-quasi-fold change threshold. b) Top 250 significantly DMRs (q<0.05) are highlighted in with black dots with all other DMRs shown in gray dots. These DMRs were used for the focused analysis in Figure 2b.

**Additional file5**:** Figure S7**. Arbitrary set of five patient sample copy number variation from CNF and PNFs demonstrate focal amplifications and deletions. A set of five patient samples from the CNF and PNF lesion groups are plotted to show focal amplifications and deletions in both sample groups. Each dot corresponds to a 50kb bin and blue lines are segments across multiple bins to indicate larger scale amplifications and deletions. Scale is log2 relative to normal tissue types: CNF relative to normal skin; PNF relative to normal nerve. All data were analyzed using SeSAMe.

**Additional file6**: **Table S1**. …

## Data Availability

All data generated or analyzed during this study are included in this published article and its supplementary information files or are available from the corresponding author upon reasonable request. Statistical Source Data underlying all figures are provided as a separate file with a tab for each panel and the unprocessed western blots as Source Data. A Nature Research Reporting Summary for this article is available as a Supplementary Information file.
