# Supplementary material for: Distinctive epigenomic alterations in NF1-deficient cutaneous and plexiform neurofibromas drive differential MKK/p38 signaling
Source: Epigenetics Chromatin. 2021 Jan 13;14:7. doi: 10.1186/s13072-020-00380-6 (PMC7805211; doi:10.1186/s13072-020-00380-6)
Supplement: Supplementary file 2 — Additional file2: Figure S4. Raw and normalized beta value densities from Illumina EPIC methylation arrays across patients and sample types. a) Raw beta value density plots following removal of failed samples and low-quality and cross-reactive probes (see Methods). b) Normalized beta value density plots following single-sample normalization (ssNOOB) reduces per-sample technical variation that can skew downstream analyses. [file 13072_2020_380_MOESM2_ESM.docx]

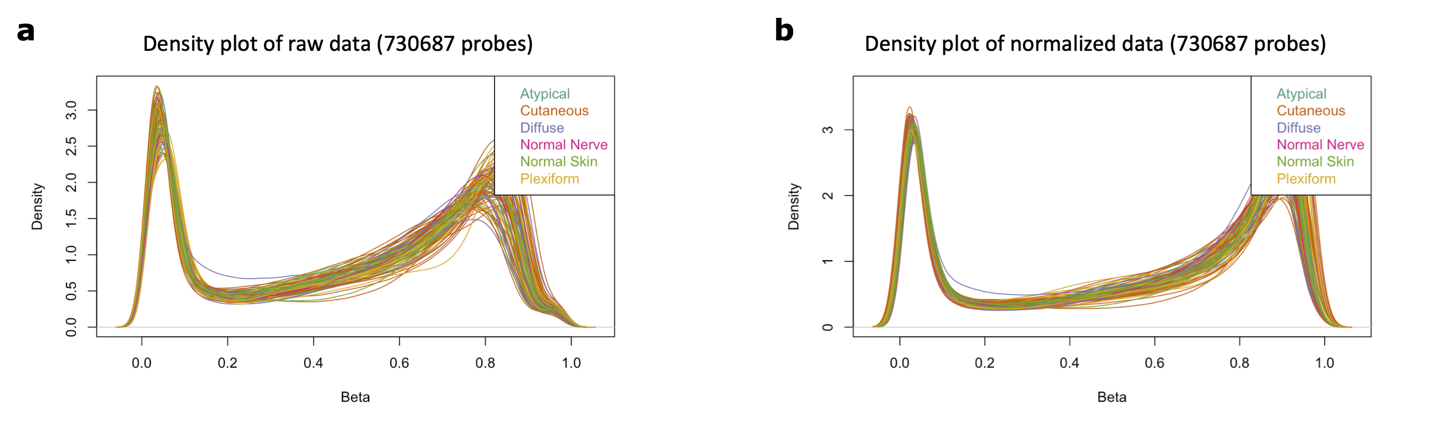


**Supplemental Figure 4. Raw and normalized beta value densities from Illumina EPIC methylation arrays across patients and sample types.** a) Raw beta value density plots following removal of failed samples and low-quality and cross-reactive probes (see Methods). b) Normalized beta value density plots following single-sample normalization (ssNOOB) reduces per-sample technical variation that can skew downstream analyses.
