# Supplementary material for: Distinctive epigenomic alterations in NF1-deficient cutaneous and plexiform neurofibromas drive differential MKK/p38 signaling
Source: Epigenetics Chromatin. 2021 Jan 13;14:7. doi: 10.1186/s13072-020-00380-6 (PMC7805211; doi:10.1186/s13072-020-00380-6)
Supplement: Supplementary file 3 — Additional file3: Figure S5. Singular value decomposition analysis of technical and biological sources of variation. Detection of sources of technical variation that need to be accounted for in the differential methylation models and adjusted for prior to visualization was accomplished using the champ.SVD function implemented in ChAMP. Technical factors such as slide and scan date were found to contribute significant sources of variation in the data in addition to biological factors, such as sex, that needed to be added as covariates in the differential methylation models to test for sample group differences (CNF and PNF). [file 13072_2020_380_MOESM3_ESM.docx]

**
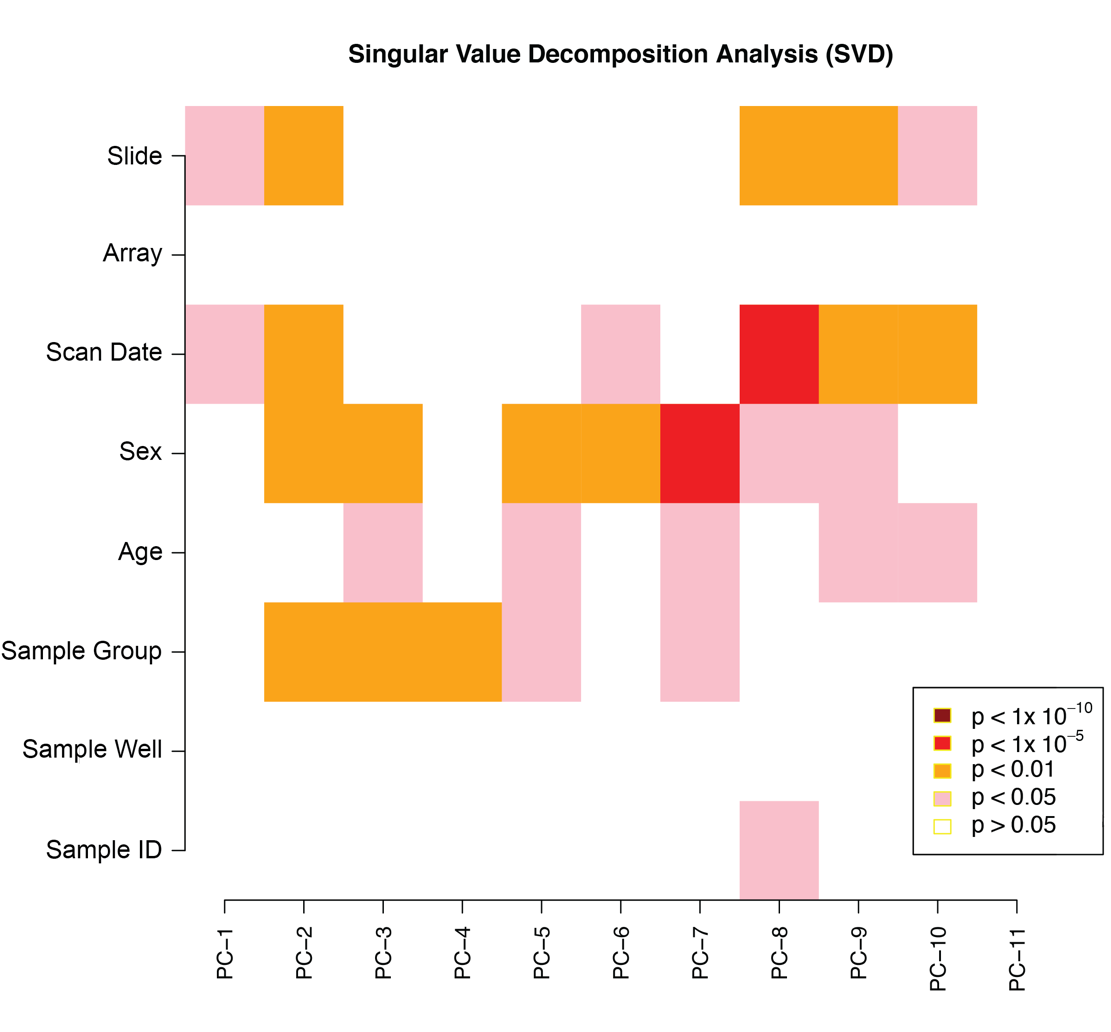
Supplemental Figure 5. Singular value decomposition analysis of technical and biological sources of variation.** Detection of sources of technical variation that need to be accounted for in the differential methylation models and adjusted for prior to visualization was accomplished using the champ.SVD function implemented in ChAMP. Technical factors such as slide and scan date were found to contribute significant sources of variation in the data in addition to biological factors, such as sex, that needed to be added as covariates in the differential methylation models to test for sample group differences (CNF and PNF).
