# Supplementary material for: Distinctive epigenomic alterations in NF1-deficient cutaneous and plexiform neurofibromas drive differential MKK/p38 signaling
Source: Epigenetics Chromatin. 2021 Jan 13;14:7. doi: 10.1186/s13072-020-00380-6 (PMC7805211; doi:10.1186/s13072-020-00380-6)
Supplement: Supplementary file 4 — Additional file4: Figure S6. Volcano plots of top significantly differentially methylated regions. a) Significantly differentially methylated regions (DMRs; q<0.05) with a log2-quasi-fold change (see Methods) greater than 1 are highlighted with black dots. Gray dots correspond to DMRs falling below the log2-quasi-fold change threshold. b) Top 250 significantly DMRs (q<0.05) are highlighted in with black dots with all other DMRs shown in gray dots. These DMRs were used for the focused analysis in Figure 2b. [file 13072_2020_380_MOESM4_ESM.docx]

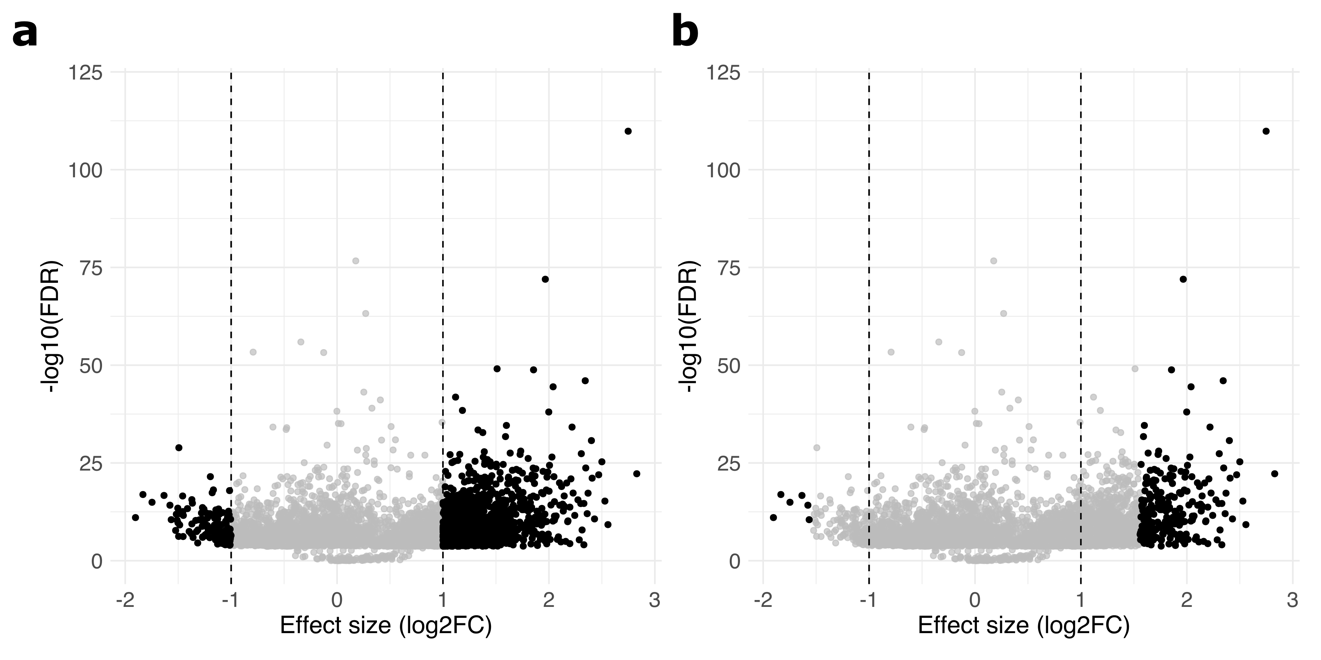


**Supplemental Figure 6. Volcano plots of top significantly differentially methylated regions.** a) Significantly differentially methylated regions (DMRs; q<0.05) with a log2-quasi-fold change (see Methods) greater than 1 are highlighted with black dots. Gray dots correspond to DMRs falling below the log2-quasi-fold change threshold. b) Top 250 significantly DMRs (q<0.05) are highlighted in with black dots with all other DMRs shown in gray dots. These DMRs were used for the focused analysis in **Figure 2b**.
