# Supplementary material for: Distinctive epigenomic alterations in NF1-deficient cutaneous and plexiform neurofibromas drive differential MKK/p38 signaling
Source: Epigenetics Chromatin. 2021 Jan 13;14:7. doi: 10.1186/s13072-020-00380-6 (PMC7805211; doi:10.1186/s13072-020-00380-6)
Supplement: Supplementary file 5 — Additional file5: Figure S7. Arbitrary set of five patient sample copy number variation from CNF and PNFs demonstrate focal amplifications and deletions. A set of five patient samples from the CNF and PNF lesion groups are plotted to show focal amplifications and deletions in both sample groups. Each dot corresponds to a 50kb bin and blue lines are segments across multiple bins to indicate larger scale amplifications and deletions. Scale is log2 relative to normal tissue types: CNF relative to normal skin; PNF relative to normal nerve. All data were analyzed using SeSAMe. [file 13072_2020_380_MOESM5_ESM.docx]

**
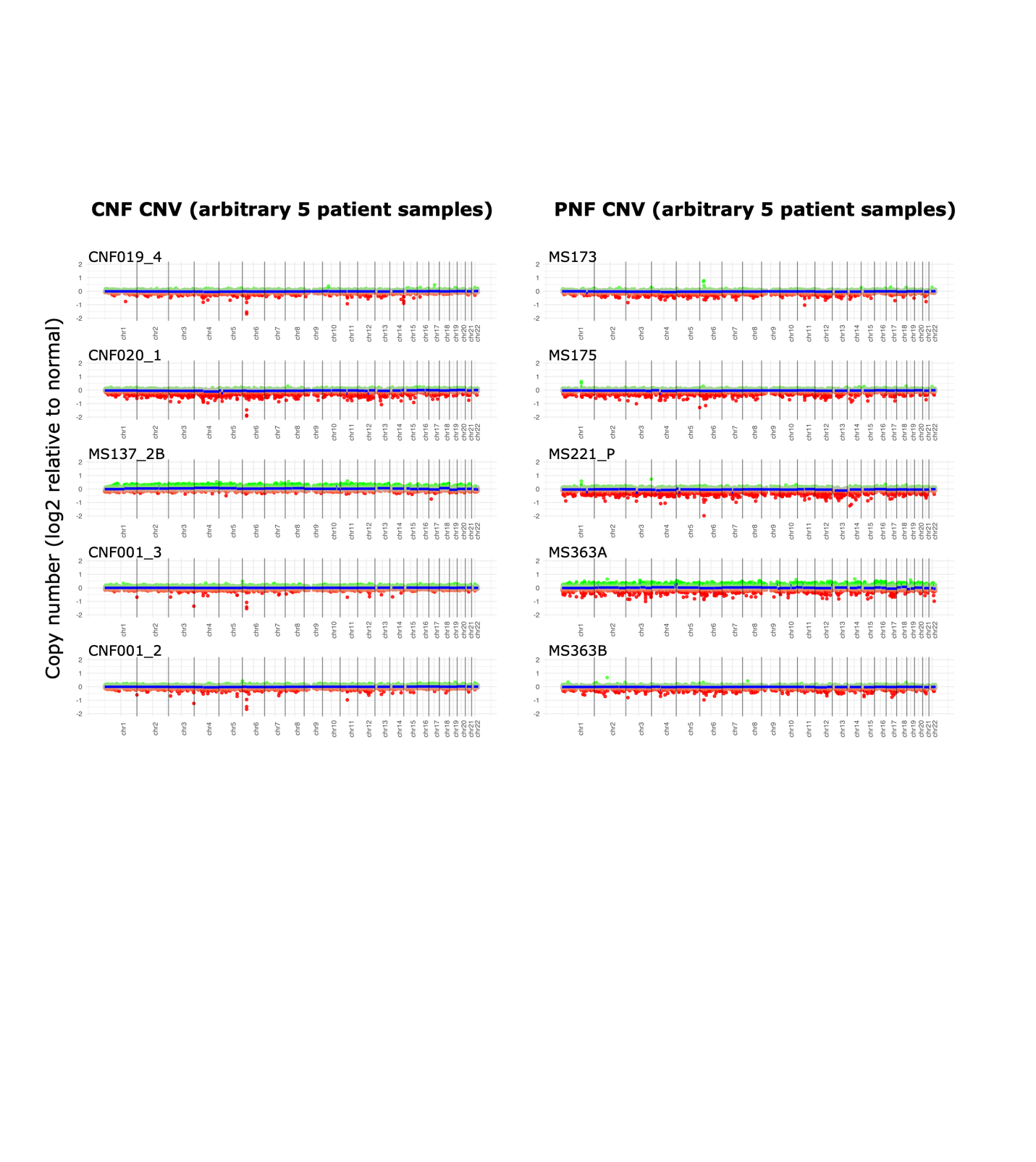
**

**Supplemental Figure 7. Arbitrary set of five patient sample copy number variation from CNF and PNFs demonstrate focal amplifications and deletions.** A set of five patient samples from the CNF and PNF lesion groups are plotted to show focal amplifications and deletions in both sample groups. Each dot corresponds to a 50kb bin and blue lines are segments across multiple bins to indicate larger scale amplifications and deletions. Scale is log2 relative to normal tissue types: CNF relative to normal skin; PNF relative to normal nerve. All data were analyzed using SeSAMe.
