# Supplementary figures and images for: Distinctive epigenomic alterations in NF1-deficient cutaneous and plexiform neurofibromas drive differential MKK/p38 signaling
Source: Epigenetics Chromatin. 2021 Jan 13;14:7. doi: 10.1186/s13072-020-00380-6 (PMC7805211; doi:10.1186/s13072-020-00380-6)

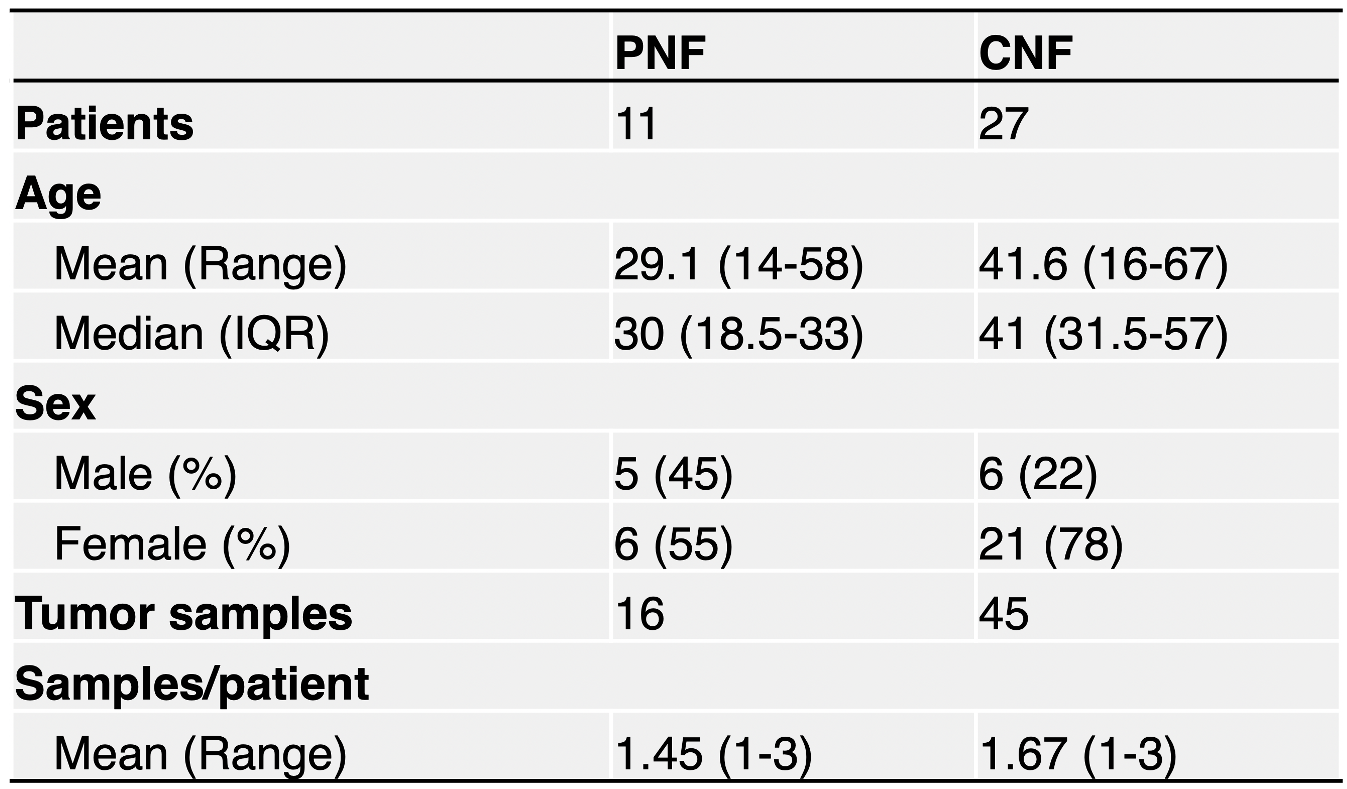

Supplement: Supplementary file 6 — Additional file6: Table S1. … [file 13072_2020_380_MOESM6_ESM.png]
